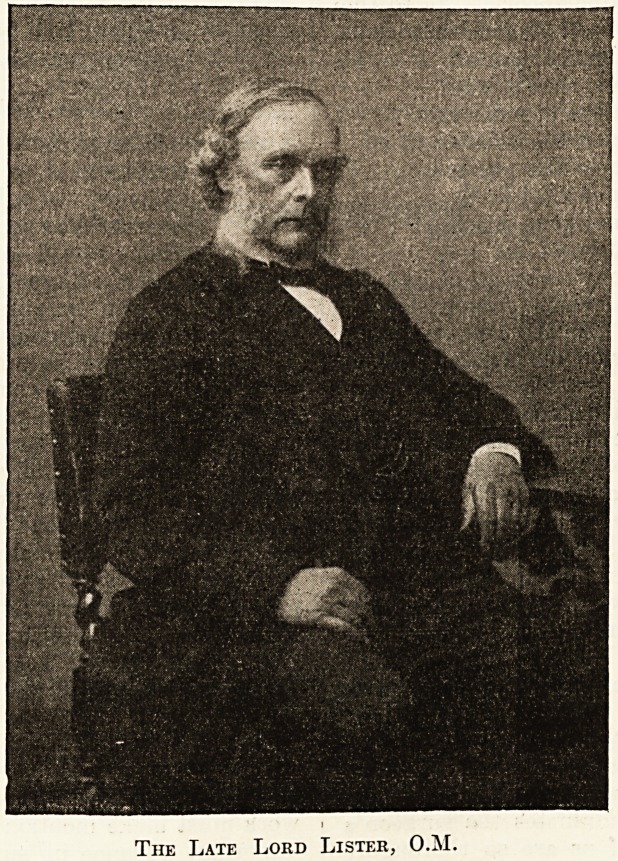# The Greatest of the Victorians

**Published:** 1912-02-17

**Authors:** 


### THE GREATEST OF THE VICTORIANS.

### Lord Lister's Achievement.

Throughout the hospitals of the entire world there can be but one common and prevailing topic this week, and one unanimous sentiment. The death of Lord Lister, full of years and honours, must unite us all in homage to his greatness and in tributes of affectionate gratitude to his memory. If tuberculosis be indeed, as it has been named, the Captain of the Men of Death, surely Lister was the Captain of the Men of Life; for it was computed in 1900, when the nineteenth century came to its close, that Lister's work had already then saved more lives than were sacrificed in the whole of the devastating wars of that contentious century. Not only the hospital world, but the whole world, from the highest to the lowest, ought to pay the last honour to Lord Lister; for his work has benefited the poor and outcast not less but more than the wealthy and high-placed. And that work is going on still, and doubtless will continue, so that posterity will revere his name and honour his memory more and more as the years roll by.

The full import of Lister's life and works can perhaps hardly be appreciated by those who have been surgically brought up during the last thirty years. The horrors of the old-time hospital wards and of the old-time surgery were familiar enough to many who are still in harness, and scarcely think of themselves yet as veterans; but to the majority they are merely visions of a dream, and somewhat misty visions at that. A glance through the surgical registers of forty or fifty years ago at any large hospital helps a little to a comprehension of what pre-Listerian surgery meant. Modern surgery at every step is built upon Lister's solid foundation; were that disturbed, the whole superstructure would fall in ruins to the ground.

The Late Lord Lister, O.M.

A comprehensive survey of Lister's achievements would fill many pages of this journal. But it is not unprofitable to seek for the lessons which those achievements teach. The two essential characteristics of the man in all phases of his life were simplicity and honesty. Lister was honest with himself, and that trait was a valuable asset to him always. Whenever an unexpected failure seemed to vitiate his scheme, he would always cast about for the source of error and set to work to eliminate it. Lister was never the man to put blame on a subordinate for any unexplained disaster. He was never afraid to admit a mistake; and no better instance can be adduced than the readiness with which he gave up the carbolic spray as soon as he felt convinced that his critics were right about it. Lister, in fact, was great enough not to harbour pettiness of any kind; though the pettiness of others was only too often displayed against him.

Then again, Lister was never content with success, admitted and patent. The artist's spirit urged him continually onwards to further approaches towards perfection; long after he had shown the world how to make wounds heal, he was always hard at work perfecting his technique, always a lap ahead of the nearest competitor. Granted that Lister was unfortunate in living when no one had thought of applying the discoveries of Pasteur to surgery, and when Semmelweis and Wendell Holmes had shown the value of cleanliness, yet Lister showed true genius in his first concept of antisepsis and in the splendid assiduity with which he worked it out. It is often carelessly supposed that a flash of inspiration put Lister on the right track; whereas in truth Lister was a first-rate surgeon, an original thinker, and a patient researcher before his first tentative efforts at antiseptic treatment. Inspiration, no doubt, was his; but so it was also with two or three eighteenth-century surgeons, who got so extraordinarily near the truth that one realises, when reading their writings, just how supreme the genius of Lister was. Yet another prominent feature was his imperturbability. For some time he was publicly derided as well as privately criticised; but Lister was serene in the conviction that time and he must triumph, and he could afford to proceed calmly with his work, and to ignore those whose jeers at last recoiled heavily upon their own heads.

To know that Lister was an Englishman is to make one proud to be English too. Yet Lister's discoveries are international and world-wide in their application. He "was not for an age, but for all time"; neither was he for one country, but for all the sick and suffering of every nation and every time; and he was the greatest of the many great men who shed such lustre upon the Victorian era.

Lister's claim to originality is not lessened when we examine the share which his predecessors and coadjutants had in that general preparation of mind which always seems to make itself felt before the advent of an original scientific theory. As to the discovery of the antiseptic method for instance it is well known, and Lister himself insisted on the recognition of the fact, that it was the researches of Pasteur on the processes of fermentation which directed the attention of the Glasgow surgeon to the possibility that the decomposition of vital fluids might be explained in an analogous manner. That this was the source of his inspiration does not detract from the credit of Lister's work, for something like a whole decade elapsed between the publication of Pasteur's experiments and the first paper by Lister on the new method of treating compound fractures, and it was open to any other surgeon to have anticipated Lister in this direction. But it must be borne in mind that Lister's work was not of the sudden-flash-of-inspiration order. If ever a man could be said to have prepared himself by a thorough scientific training and by patient toil, that man was Lister, and probably with a share of his for their predilection. From the earliest days of his medical career he had devoted himself to microscopy in the fields of histology and pathology, having convinced himself that in the elucidation of pathological problems lay the brightest hope for the future of surgery. Even with the lead given by Pasteur's discoveries it still required a man with a keen insight to follow these flickerings of light and to fan them into the steady flame which now illuminates the surgeon's path, and to which most of us are so thoroughly accustomed that we fail to realise the horror of the pre-existing gloom.

Much careful work was done before Lister published in 1867 his first paper, and addressed, in the same year, the meeting of the British Medical Association in Dublin, on the subject of the antiseptic method. Perhaps the statement that appealed most to his audience on that occasion was the announcement that for nine months no case of pyæmia or of hospital gangrene had occurred in Lister's wards at Glasgow.

As we have said, fully to appreciate the effect of this statement one needs to have been in hospital practice more than forty years ago, and we may here call attention to a point that is sometimes overlooked. The amount of suppuration, pyæmia, and gangrene in hospitals in the sixties was greater than, say, in the forties, for the reason that another great beneficent discovery in surgery, namely, chloroform anæsthesia, had enabled the surgeons of Lister's time to perform more extensive and more numerous operations than had hitherto been possible, and thus the number of wounds made was far greater. The patients exchanged their early torturings for the deferred sufferings of a prolonged convalescence.

As is now well known the victory for antisepsis was not gained forthwith; there followed for some years keen criticism and scoffing; the very modifications and improvements introduced by Lister and his pupils were made the excuse for decrying his methods; but now all humanity combines in honouring the man who first pointed to the practical abolition of the frightful scourge of post-operative sepsis, and which has led to the present simpler aseptic methods.

### The Work of Semmelweis.

Though Lord Lister has indeed been the recipient of a very large number of honours, not only in this country but also from learned societies and universities the whole world over, it has never been suggested that these rewards were commensurate with the benefits his achievements have conferred upon mankind.

It is interesting at this juncture to recall how pathetic was the contrast in the case of another pioneer in the fight against sepsis. We refer, of course, to the brilliant Semmelweis, who anticipated in the maternity wards of the Vienna hospitals some of the principles of asepsis which were later elaborated independently by Lister. Unfortunately Semmelweis in his successful attempts to reduce the appalling mortality of lying-in cases from puerperal fever encountered strong opposition and prejudice, not to say contumely, and died broken-hearted before his really magnificent efforts were accorded their due.

### Catgut Ligatures.

Even the briefest references to Lord Lister's work must make mention of another almost equally important advance in surgery which would alone have made his name. The introduction of the absorbable ligature of catgut was an improvement which had far-reaching consequences, and was again due to the same master mind backed, be it not forgotten, by a long and careful training in pathological research. Instead of leaving bunches of silk or thread ligatures hanging out of an open wound till they fell off loosened by suppurative inflammation, it now became possible to close a wound and bury the ligature, thus not only saving much time, but reducing enormously the risk of blood-poisoning from the infection of a large exposed surface.

## Figures and Tables

**Figure f1:**